# Altered brain structural networks in attention deficit/hyperactivity disorder children revealed by cortical thickness

**DOI:** 10.18632/oncotarget.14734

**Published:** 2017-01-18

**Authors:** Tian Liu, Yanni Chen, Chenxi Li, Youjun Li, Jue Wang

**Affiliations:** ^1^ The Key Laboratory of Biomedical Information Engineering of Ministry of Education, Institute of Biomedical Engineering, School of Life Science and Technology, Xi’an Jiaotong University, Xi’an, P. R. China; ^2^ National Engineering Research Center of Health Care and Medical Devices, Xi’an Jiaotong University Branch, Xi’an, P. R. China; ^3^ Xi’an Children’s Hospital, Xi’an, P. R. China; ^4^ The First Affiliated Hospital of Xi’an Jiaotong University, Xi’an, P. R. China

**Keywords:** attention deficit/hyperactivity disorder, cortical thickness, magnetic resonance imaging, small-world, structural networks

## Abstract

This study investigated the cortical thickness and topological features of human brain anatomical networks related to attention deficit/hyperactivity disorder. Data were collected from 40 attention deficit/hyperactivity disorder children and 40 normal control children. Interregional correlation matrices were established by calculating the correlations of cortical thickness between all pairs of cortical regions (68 regions) of the whole brain. Further thresholds were applied to create binary matrices to construct a series of undirected and unweighted graphs, and global, local, and nodal efficiencies were computed as a function of the network cost. These experimental results revealed abnormal cortical thickness and correlations in attention deficit/hyperactivity disorder, and showed that the brain structural networks of attention deficit/hyperactivity disorder subjects had inefficient small-world topological features. Furthermore, their topological properties were altered abnormally. In particular, decreased global efficiency combined with increased local efficiency in attention deficit/hyperactivity disorder children led to a disorder-related shift of the network topological structure toward regular networks. In addition, nodal efficiency, cortical thickness, and correlation analyses revealed that several brain regions were altered in attention deficit/hyperactivity disorder patients. These findings are in accordance with a hypothesis of dysfunctional integration and segregation of the brain in patients with attention deficit/hyperactivity disorder and provide further evidence of brain dysfunction in attention deficit/hyperactivity disorder patients by observing cortical thickness on magnetic resonance imaging.

## INTRODUCTION

Attention deficit/hyperactivity disorder (ADHD) is one of the most common childhood behavioral disorders. Subjects who are diagnosed with ADHD are usually hyperactive, impulsive, and inattentive [1]. There are approximately 5 – 8% ADHD patients worldwide. Although ADHD is a common childhood neurodevelopmental disorder, it usually continues into adulthood [2].

Many neuropsychological and neuroanatomical studies have demonstrated that ADHD symptoms are associated with functional [3, 4] and anatomical [5, 6] abnormalities including various neural regions: cerebellum, ventrolateral prefrontal cortex, dorsolateral prefrontal cortex, striatum, and parietal cortex. These cortical regions are the main components of the cognition and attention parallel networks [1]. Increasing numbers of studies of ADHD have recently focused on brain network disorders instead of regional brain changes.

The continuous development of graph theory methods has allowed investigators to determine the topological features of complex brain networks. Watts and Strogatz [7] first defined “small-world networks” as graphs with a few random long-distance connections and many local connections, which were indicative of near-optimum structural networks. Since then, complex brain networks have become a hot spot in neuroscience [8–11]. Many recent studies have reported that the small-world features of brain networks were altered in brain diseases such as epilepsy [12], Alzheimer's disease [13], spinal core injury [14], schizophrenia [15], and brain tumor [16]. Increasing evidence has also demonstrated that ADHD is closely related to brain function network abnormalities [17, 18]. However, to date, the underlying architecture of the brain anatomical network in ADHD patients is still poorly understood. Study of this network is necessary to reveal how the brain function of ADHD subjects is related to their structural substrates from intrinsically structural organizational principles in the human brain. In the present study, we hypothesized that the topological organization of brain structural networks was altered in ADHD children.

To test this hypothesis, morphological measurements by *in vivo* magnetic resonance imaging (MRI) were used to construct large-scale anatomical networks of the brain of both ADHD and control children. Cortical thickness was selected as a morphometric characteristic because it reflects both mental and neurological disorders as well as normal development. First, we measured the thickness of the gray matter of the human cerebral cortex using computational neuroanatomy and segmented the whole cerebral region into 68 areas. Then, we established and analyzed a set of correlation matrices by calculating the correlations of cortical thickness between all pairs of the cortical cortex of the whole brain. The resulting correlation matrices were subjected to further thresholds to create a binary matrix to construct an undirected and unweighted graph. Then, the topological features of the graph were computed by graph theoretical analysis. Finally, we analyzed the correlations between the average cortex thickness and clinical symptoms of ADHD.

## RESULTS

### Cortical thickness differences

The present study used ANOVA to investigate the difference in cortical thickness between the two groups. As shown in Table 2, we observed several abnormal cortical thicknesses concentrated in the frontal and temporal regions in ADHD patients. Many brain regions showed significant ADHD-associated thinning, especially in some bilateral homologous regions. For example, decreased bilateral hemisphere cortical thickness appeared in the pars orbitalis, caudal middle frontal, lateral orbitofrontal, fusiform, inferior temporal, pars opercularis, pars triangularis, superior frontal, rostral middle frontal, and temporal pole in the ADHD group. In addition, ADHD patients also had a reduced thickness in entorhinal, inferior parietal region of the left hemisphere, and medial orbitofrontal, precentral region of the right hemisphere, respectively.

**Table 1:** Group characteristics

	ADHD (*n* = 40)	Control (*n* = 40)	*P* Value
Age (years)	12.47 ± 2.01	11.76 ± 1.75	0.02
Gender (M/F)	40/0	40/0	
Full IQ	110.57 ± 13.42	115.76 ± 13.53	0.067

Values are presented as the mean ± SD; Abbreviations: M: male, F: female; Independent t-test.

**Table 2:** Significant cortical thinning regions of the brain in ADHD patients compared with controls

Region	Mean (standard error)		F _1, 80_ value	*P* value
	ADHD	NC		
Caudal middle frontal. L	2.7876(0.01596)	2.8372(0.01679)	4.540	0.035
Entorhinal. L	3.4096(0.04336)	3.5744(0.04161)	7.518	0.007
Fusiform. L	2.8907(0.02055)	2.9682(0.02042)	7.131	0.008
Inferior parietal. L	2.7725(0.01801)	2.8266(0.01679)	4.848	0.029
Inferior temporal. L	2.9804(0.02351)	3.0675(0.02568)	6.192	0.014
Lateral orbitofrontal. L	2.9607(0.02510)	3.0509(0.02071)	7.784	0.006
Pars opercularis. L	2.8211(0.01702)	2.8954(0.01759)	9.144	0.003
Pars orbitalis. L	2.9883(0.03099)	3.0889(0.02737)	5.975	0.016
Pars triangularis. L	2.7925(0.01933)	2.8531(0.02021)	4.657	0.032
Rostral middle frontal. L	2.6494(0.01902)	2.7393(0.01686)	12.567	0.001
Superior frontal. L	3.0709(0.01604)	3.1496(0.01631)	11.757	0.001
Temporal pole. L	3.6461(0.04065)	3.7937(0.02991)	8.752	0.004
Caudal middle frontal. R	2.7539(0.01681)	2.8220(0.01858)	7.276	0.008
Fusiform. R	2.9273(0.02133)	2.9976(0.02139)	5.394	0.021
Inferior temporal. R	3.0389(0.02238)	3.1484(0.02310)	11.513	0.001
Lateral orbitofrontal. R	2.8731(0.02366)	2.9793(0.02029)	11.732	0.001
Medial orbitofrontal. R	2.7169(0.02709)	2.8192(0.01969)	9.544	0.002
Pars orbitalis. R	2.9479(0.02420)	3.0620(0.02760)	9.507	0.002
Pars triangularis. R	2.7621(0.01954)	2.8282(0.01674)	6.678	0.011
Precentral. R	2.6211(0.01918)	2.6836(0.01757)	5.799	0.017
Rostral middle frontal. R	2.6064(0.01814)	2.6708(0.01809)	6.289	0.013
Superior frontal. R	2.9713(0.01700)	3.0689(0.01673)	16.696	0.000
Temporal pole. R	3.7414(0.04008)	3.8985(0.03145)	9.684	0.002

Abbreviations: NC, normal control; L, left; R, right.

### Correlations of cortical thickness between brain regions

**Figure 1:**
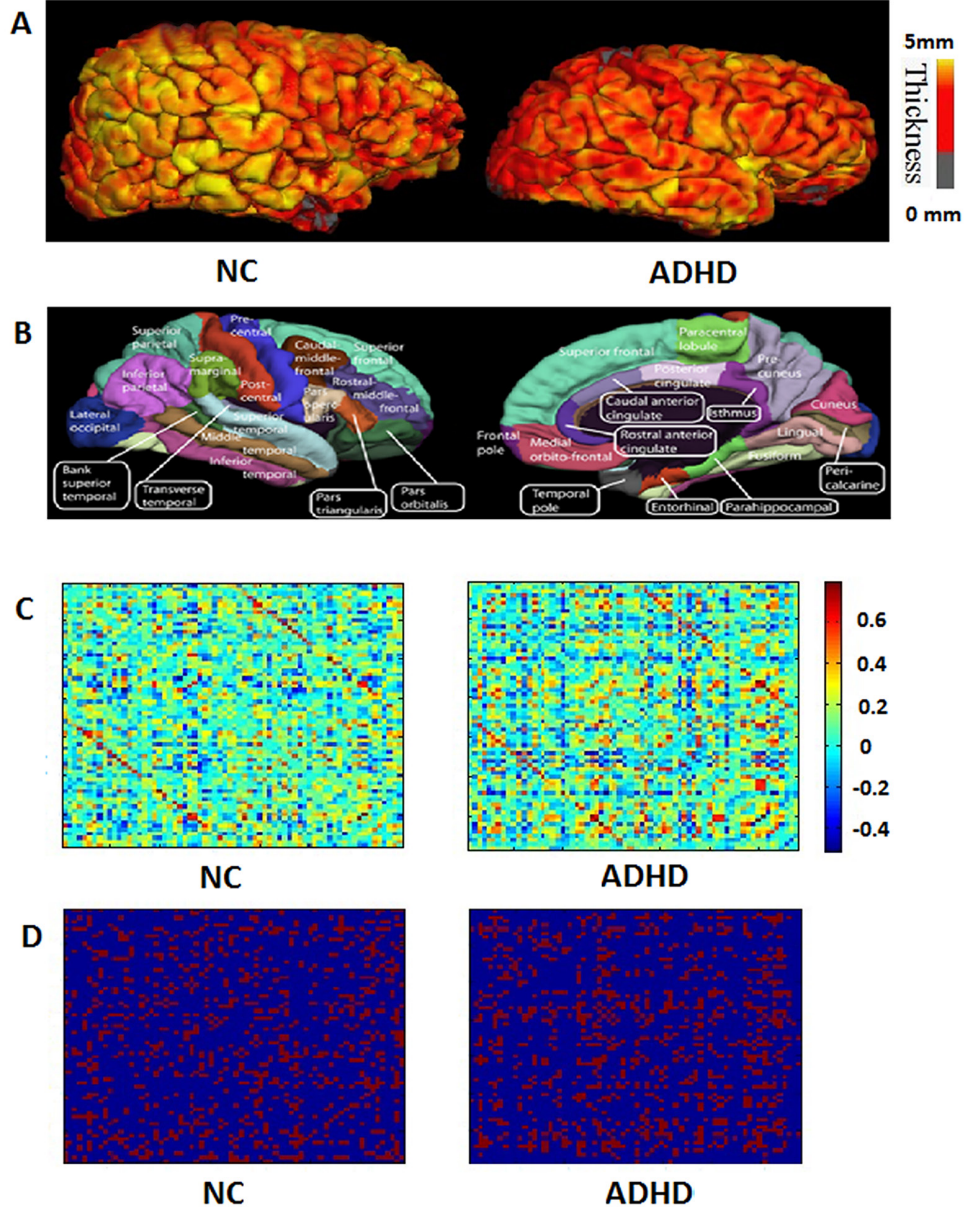
The flowchart illustrates the construction of structural cortical networks **A**. Two representative cortical thickness maps (left: normal control subject; right: ADHD subject). The color bar indicates the range of thickness. **B**. The cerebral cortex of subjects was parcellated into 68 cortical regions (left: lateral surface; right: medial surface), and each color indicates an individual region. **C**. The symmetric correlation matrix (68 × 68) was obtained by computing Pearson's correlation coefficients between the thickness of each possible pair of the 68 regions for each subject (left: normal control subject; right: ADHD patient). The color bar indicates Pearson's correlation coefficients between regions. **D**. The correlation matrix of C was thresholded into a binarized matrix (left: normal control subject; right: ADHD patient) by a cost threshold.

Figure 1C shows the cortical thickness correlation coefficient matrices of the ADHD and normal controls groups. Combined with statistical analysis, the present study found a significant correlation in differences between the two groups in different pairs of Regions of Interest (ROIs) (Table 3). For example, increased positive correlations between the left entorhinal and left fusiform, between the left caudal anterior cingulate and right isthmus cingulate, between the left caudal anterior cingulate and right medial orbitofrontal, and between the right fusiform and right rostral anterior cingulate were observed in the ADHD patients compared with the controls. Additionally, in the ADHD subjects, we also noticed several decreased positive correlations, which were involved in the left precuneus, right caudal anterior cingulate, right caudal anterior cingulate, and right superior parietal regions (Table 3).

**Table 3:** Significant interregional cortical correlations in ADHD patients compared with controls

Region	Region	Correlation, r		Z score
		ADHD	NC	
**Increased positive correlations in ADHD**				
Left entorhinal	Left fusiform	0.72	0.39	5.09
Left caudal anterior cingulate	Right isthmus cingulate	0.42	-0.04	4.25
Left caudal anterior cingulate	Right medial orbitofrontal	0.53	0.09	4.15
Right fusiform	Right rostral anterior cingulate	0.53	0.16	4.45
**Decreased positive correlations in ADHD**				
Left precuneus	Right caudal anterior cingulate	-0.09	0.34	4.96
Right caudal anterior cingulate	Right superior parietal	-0.21	0.27	4.53

The *r* values are indicative of correlation coefficients of cortical thickness between regions in the ADHD patients or normal controls (NC). *Z* scores are the results of *Z* statistics. All listed *Z* scores in this work are significant (*P* < 0.05, FDR-corrected).

### Small-world features of structural networks

**Figure 2:**
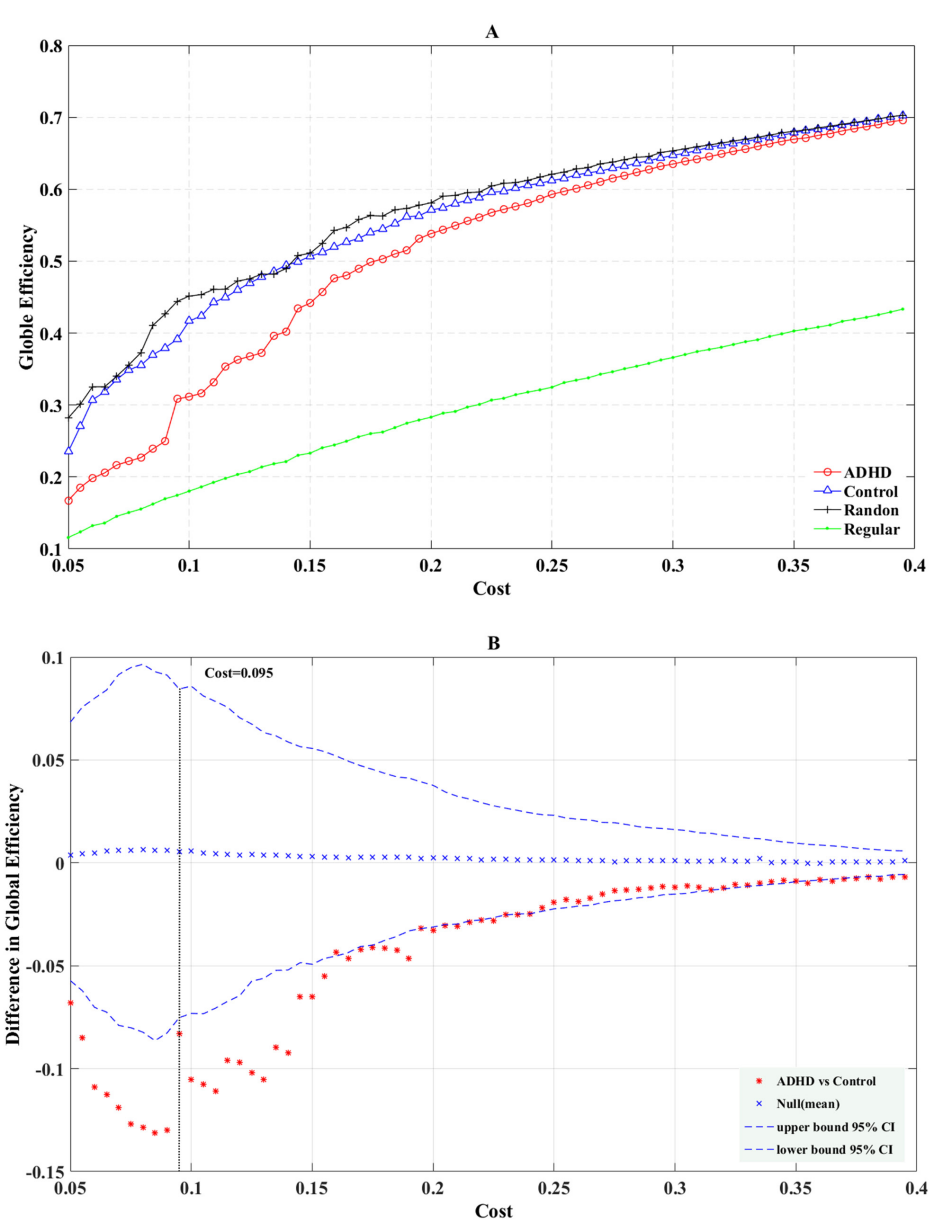
The global (A) efficiency is shown as a function of the cost for random (crosses), regular (points), ADHD (circles), and control (triangles) brain networks The permutation test results of global efficiency are shown in B. The asterisks indicate differences of global (B) efficiency between ADHD and control groups; crosses indicate mean differences of global (B) efficiency; curve of dashes indicate the upper and lower boundaries of the 95% confidence interval.

Figure 2 illustrates the global efficiency computed in the regular, random, and real structural networks of ADHD and normal control groups as the function of cost. We found that the global efficiency of the two groups were ascendant as the cost rose, and was intermediate between the regular and random networks (Figure 2A). The permutation test results of global efficiency (Figure 2B) showed that at all ranges of cost values, the structural networks of the ADHD group manifested declining global efficiency (the difference between ADHD *E*glob *(G)* and the control *E*glob *(G)* was lower than the mean difference value of global efficiency). For the range of costs (0.05–0.25), *E*glob *(G)* was significantly lower in the ADHD group than the normal control group (*P* < 0.05), and when the cost was 0.06, the difference between the two groups was the most significant (*P* = 0.001). For the range of costs (0.26–0.4), the trend curve was almost consistent with the lower boundary of the 95% confidence interval.

**Figure 3:**
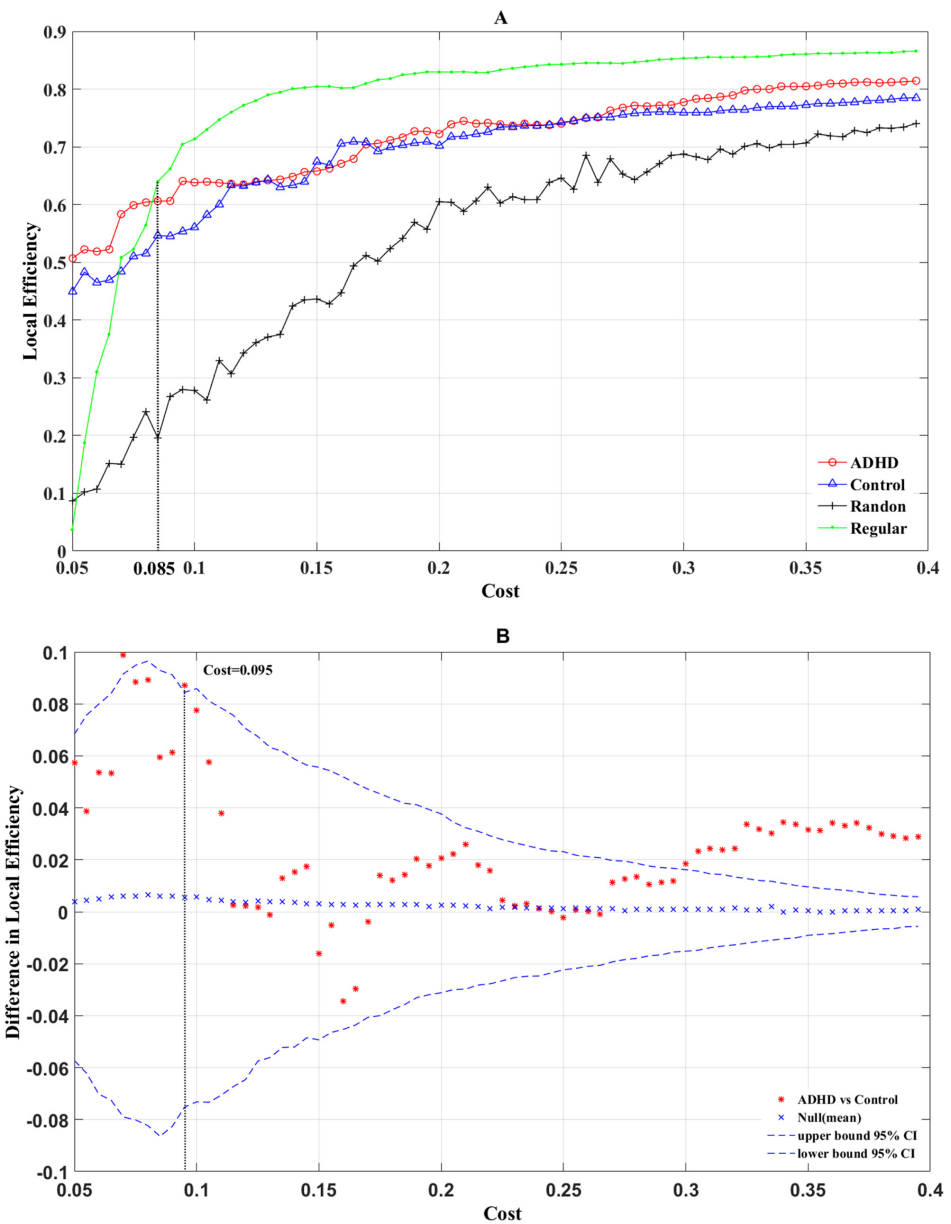
The local (A) efficiency is shown as a function of the cost for random (crosses), regular (points), ADHD (circles), and control (triangles) brain networks The permutation test results of local efficiency are shown in B. The asterisks indicate differences of local (B) efficiency between ADHD and control groups; crosses indicate mean differences of local (B) efficiency; curve of dashes indicate the upper and lower boundaries of the 95% confidence interval.

Additionally, we also observed that the local efficiency of the real networks of the two groups was enhanced with an increase in cost. However, unlike global efficiency, the local efficiency of the real brain networks was larger than that of the random network and was lower than that of the regular network for a cost > 0.085 (Figure 3A). The permutation test results of local efficiency (Figure 3B) showed that at most range of cost values, the structural networks of the ADHD group showed an increased local efficiency (the difference between the ADHD *E*loc *(G)* and the control *E*loc *(G)* was higher than the mean difference value of local efficiency). For the range of costs (0.31–0.4), *E*loc *(G)* was significantly greater for the ADHD group compared with the control group (*P* < 0.05), and at a cost of 0.375, the most significant between-group difference (*P* = 0.0342) was observed.

**Figure 4:**
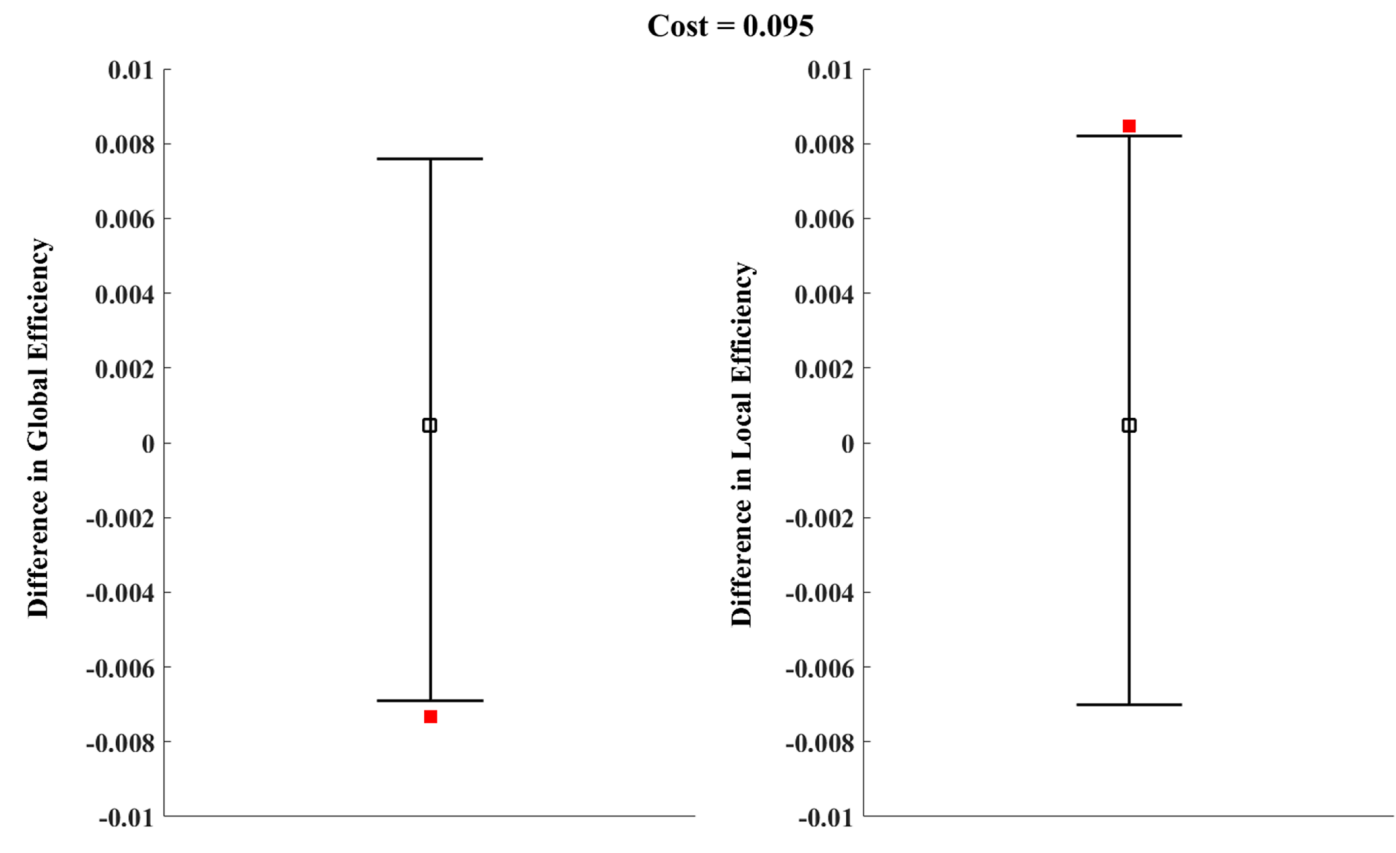
The permutation test results of global and local efficiency at a cost of 0.095 Error bars indicate the upper and lower boundaries of the 95% confidence interval; hollow squares indicate mean differences of global (left) or local (right) efficiency; and filled squares indicate differences of global (left) or local (right) efficiency between ADHD and control groups.

Previous results demonstrated that costs of 0.06 and 0.375 produced the most significant differences between the two groups. Therefore, a comparison of the global efficiency and local efficiency of real brain networks in cost = 0.06 and cost = 0.375, respectively, was made. When cost = 0.06, there was no significant between-group difference for *E*loc (*G*) (Figure 3B, *P* = 0.072). In addition, the local efficiency of the real brain networks were larger than the regular brain networks for a cost < 0.085 (Figure 3A), which indicated that for cost < 0.085, the real brain networks could not constitute a small-world network. Similarly, when cost = 0.375, the *E*glob *(G)* of the ADHD group was significantly lower than in the control group (Figure 2B, *P* = 0.041) and the *E*loc *(G)* of the ADHD group was significantly higher than in the control group (Figure 3B, *P* = 0.034). These results suggested the structural networks of the two groups possessed small-world architecture when cost = 0.375. However, previous studies posited that the brain network is economical and efficient with a comparatively low cost [36]. Thus, a cost of 0.375 may be not true for real brain networks. According to the above results, we chose a cost of 0.095 to compare the *E*glob and *E*loc again (Figure 4). As expected, the *E*glob (*G*) of the ADHD group was significantly lower than in the control group (Figure 4 left, *P* = 0.04) and the *E*loc (*G*) of the ADHD group was significantly higher than in the control group (Figure 4 right, *P* = 0.047). Under this cost value, both networks manifested small-world characteristics for their global and local efficiency when compared with the matched regular and random networks (Figures 2A, 3A). Consequently, the present study tested the group difference of nodal efficiency at a cost of 0.095.

**Figure 5:**
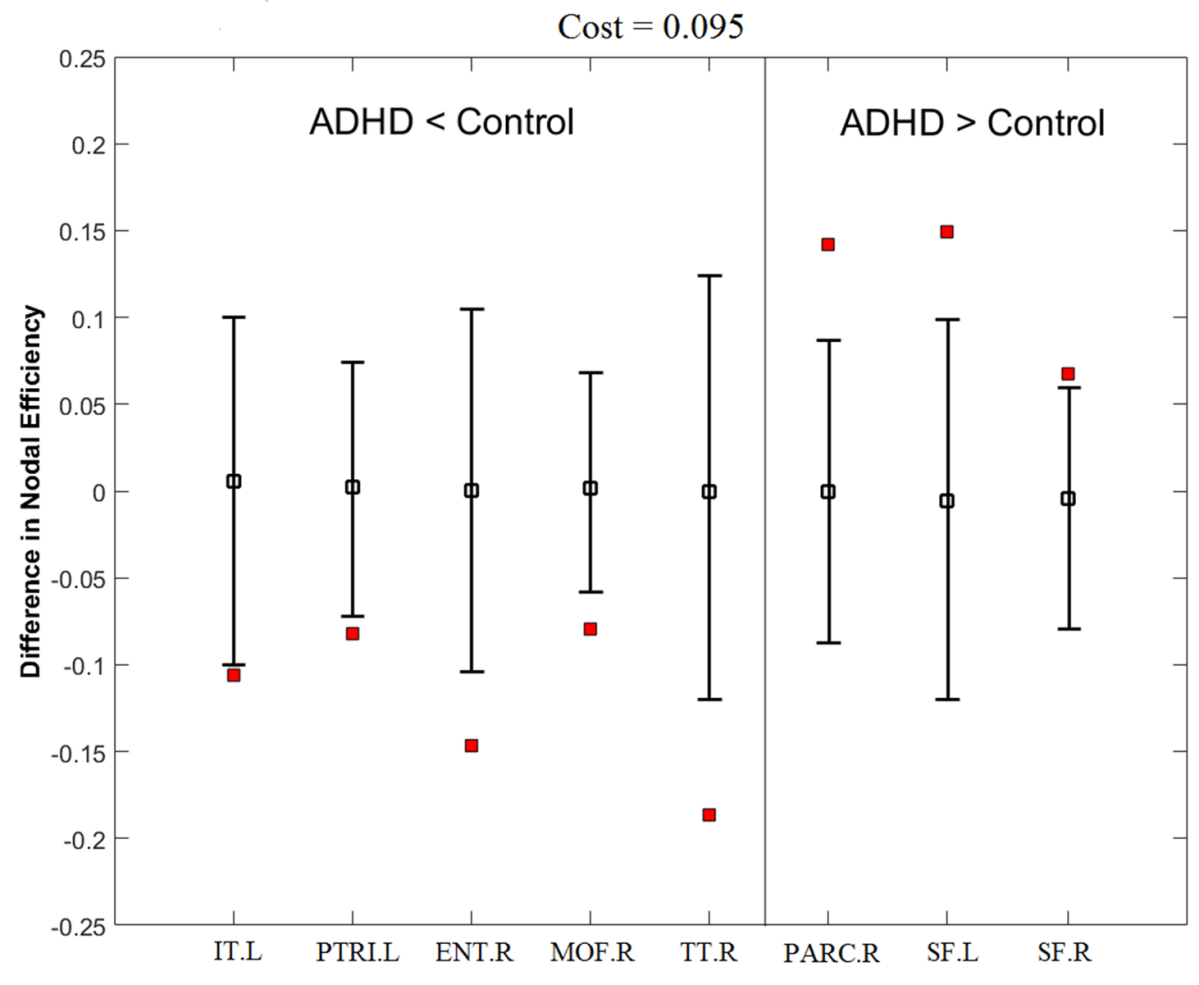
ADHD related changes in nodal efficiency at a cost of 0.095 Error bars indicate the upper and lower boundaries of the 95% confidence interval. The hollow squares and filled squares correspond to the mean differences of nodal efficiency and differences of nodal efficiency between ADHD and control groups, respectively. See Table 5 for details of the regions. See Appendix for abbreviations of the regions.

Nodal efficiency reflects the effect of the disorder on the local nodal features of the brain networks. Figure 5 and Table 4 show that ADHD subjects had significantly decreased nodal efficiency in the left inferior temporal, left pars triangularis, right entorhinal, right medial orbitofrontal, and right transverse temporal cortex regions and significantly increased nodal efficiency in the left paracentral lobule, and bilateral superior frontal cortex regions.

**Table 4:** Regions with significant changes in nodal efficiency in ADHD patients at a cost of 0.095

Region	Hemisphere	*P* value
**Decreased nodal efficiency in ADHD**		
Inferior temporal	L	0.043
Pars triangularis	L	0.039
Entorhinal	R	0.004
Medial orbitofrontal	R	0.012
Transverse temporal	R	0.009
**Increased nodal efficiency in ADHD**		
Paracentral lobule	R	0.002
Superior frontal	L/R	0.003/0.038

Abbreviations: L, left; R, right.

**Table 5:** Correlation between the cortical thickness of left and right brain regions and ADHD_Index

Left brain regions	ADHD_Index		Left brain regions	ADHD_Index	
	*r*	Sig		*r*	Sig
Caudal anterior cingulate	-.200*	0.017	Rostral anterior cingulate	-.223**	0.007
Caudal middle frontal	-.219**	0.009	Rostral middle frontal	-.211*	0.011
Cuneus	.239**	0.004	Superior frontal	-.256**	0.002
Entorhinal	-.174*	0.037	Supramarginal	-.194*	0.02
Fusiform gyrus	-.184*	0.028	Temporal pole	-.226**	0.007
Inferior parietal	-.166*	0.048	Pars opercularis	-.190*	0.023
Inferior temporal	-.185*	0.027	Pars orbitalis	-.229**	0.006
Lateral orbitofrontal	-.198*	0.018	Pars triangularis	-.185*	0.027
Lingual gyrus	.166*	0.048	Pericalcarine	.348**	0
Middle temporal	-.172*	0.04	Precentral gyrus	-.167*	0.046
Right brain regions	ADHD_Index		Right brain regions	ADHD_Index	
	*r*	Sig		*r*	Sig
Caudal middle frontal	-.168*	0.045	Middle temporal	-.226**	0.007
Cuneus	.213*	0.011	Pars orbitalis	-.200*	0.017
Fusiform gyrus	-.189*	0.024	Pars triangularis	-.188*	0.024
Inferior temporal	-.249**	0.003	Pericalcarine	.231**	0.005
Lateral orbitofrontal	-.246**	0.003	Superior frontal	-.291**	0
Medial orbitofrontal	-.175*	0.036	Temporal pole	-.311**	0

Statistical significance (*P < 0.05; **P < 0.01, two tailed).

### Correlation analysis between ADHD clinical symptoms and cortical thickness

Correlation analyses were conducted to evaluate the relationship between cortical thickness and ADHD clinical symptom (ADHD_Index) (Table 5). The ADHD Rating Scale (ADHD-RS) IV was applied to supply dimensional measurements of ADHD symptoms. With this scale, we obtained an ADHD_Index for all subjects, which measured the overall extent of symptoms of ADHD. By calculation, we found that the ADHD_Index scores of ADHD patients were significantly correlated with the cortical thicknesses of many brain regions, such as the frontal, temporal and parietal cortex regions. In addition, most brain regions were negatively correlated with the ADHD_Index, which indicated that the smaller cortical thicknesses of these brain regions, the more obvious the symptoms would be in ADHD subjects.

## DISCUSSION

In the present study, cortical thickness data based on MRI were used to construct brain structural networks (undirected binary) for ADHD patients and normal control children. The main findings of the present research are as follows: 1) a slice of brain regions showed significant ADHD-associated thinning focused on the frontal and temporal regions; 2) abnormal cortical correlations were observed in ADHD patients compared with controls; 3) brain structural networks demonstrated small-world topology in both groups; however, ADHD patients showed altered topological properties, such as increased local efficiency and decreased global efficiency compared with the control subjects; 4) calculation of nodal efficiency demonstrated that several brain regions were altered in ADHD patients; and 5) cortical thickness of a multitude of brain regions was negatively correlated with ADHD clinical symptoms.

Given that cortical thickness describes the density, arrangement of neurons, and size, it is usually taken as suggestive of the cognitive abilities of humans [19]. Previous studies stated that cortical thinning was associated with brain disorders. Alzheimer's disease has already been characterized by cortical thinning [20], post-traumatic stress disorder (PTSD) patients exhibited a decrease in cortical thickness in some regions [21], and Parkinson disease patients show cortical thinning in the parietal and left temporal, right lateral occipital, premotor, and frontal regions [22]. Similarly, we found that several brain regions showed significant ADHD-associated thinning, mainly focused in the frontal and temporal regions (Table 2), which is in accordance with other cortical thickness studies of ADHD [23, 24]. This result demonstrated that a pattern of cortical thinning in ADHD occurs mainly in the frontal and temporal lobe, which include critical areas related to attentional mechanisms.

Empirical research of interregional morphological covariations indicated they might be associated with definite neuroanatomical pathways in the human brain [9]. Altered coordination of the brain morphology of ADHD patients might manifest as disruption of the neuroanatomical paths of patients, which might further affect brain function. Moreover, we found that ADHD children showed altered correlations (both increased positive correlation and decreased positive correlation) in areas of the default mode network, this result is in line with the research of He [20]. The default network is an anatomically defined, specific, and interconnected brain area, which is commonly shown to be active when a person does not concentrate on the outside world [25]. Teasdale [26] highlighted the competition between outside world attention and resources for internal modes of cognition. When an external task is carried out, a focus of attention to internal psychological content might slow performance or cause mistakes in the immediate task [27]. However, Gilbert [28] noted that the default network is related to a broadly tuned form of watchfulness. According to these past evidences, we infer that abnormal cortical thickness correlations of default regions in ADHD patients might affect their attention task performance.

Since Watts and Strogatz [7] quantitatively described small world networks, research into brain function networks with small-world features have been performed effectively utilizing a variety of methods, such as Magnetoencephalography [29], Electroencephalogram [13], MRI [9], and fMRI [17]. The present study found that the structural networks in the ADHD group were different from that of the control group. These results were in agreement with a study by Wang [17] on the morphological features of the brain.

The findings of structural (corpus callosum) [30] and diffusion tensor imaging (thalamocortical fibers and corticopontine fibers) [31] indicated that ADHD is associated with a loss of long-range connections [32]. These abnormalities could interfere with the long-range information communication in the brain. In this study, the global efficiency of the ADHD group was abnormally decreased over the entire cost range, which indicated that global efficiency was influenced by a lack of long-range fiber connections [17, 33]. In contrast, long-range exchange among different regions of the brain was more effective in normal children. Therefore, the global information exchange characteristics of the brain networks were less effective in ADHD children than in normal children.

The current study also found that ADHD patients had increased local efficiency compared with normal children. The underlying mechanisms of the increased local features have been widely discussed in many brain disorder studies. For example, Fallani [14] noted that the enhanced local characteristics in spinal injury subjects might be related to functional reorganization. Latora and Marchiori [33] suggested that networks with stronger local characteristics would have better fault tolerance ability in the face of external attacks. In this work, the increased local efficiency in the ADHD group might represent a compensatory action to suppress the influence of disease on the brain networks. These findings are in line with the work of Wang [17].

The brain network abnormal conversion to a regular or random network caused by disease manifested a non-optimal organizational network. Previous studies demonstrated the structure of the small world network had higher global coordination and faster flow of information than the regular network [34, 35]. In the current study, the brain structural networks of both groups showed small-world properties; however, the enhanced local efficiency and reduced global efficiency in the ADHD group (compared to the controls) demonstrated the network of the ADHD group led to a shift in the topologic structure towards a regular network, which might reflect abnormal changes in the brain structural networks of ADHD children.

Nodal efficiency tests the degree of the node connecting with all other nodes in a graph, which represents the significance of the nodal region in the entire network [36]. This study found abnormal nodal efficiency in some brain regions in ADHD patients, mainly in the frontal and temporal regions (Figure 5 and Table 4). The orbitofrontal region is associated with the executive function network. In this study, the nodal efficiency of the medial orbitofrontal (MOF) region was significantly decreased in the ADHD group, consistent with previous studies [37–39] that confirmed cortical atrophy, and suggested dopamine receptor and neurotransmitter reduction in the orbitofrontal region cortex might cause abnormal executive function in patients with ADHD. We also found the pars triangularis inferior frontal (PTRI) cortex had significantly decreased nodal efficiency. The PTRI is part of the inferior frontal gyrus, which has a major role in the response inhibitory ability [40]. The lower nodal efficiency of the PTRI cortex in ADHD patients might indicate a dysfunction in response inhibition, which is regarded as the central defect in ADHD [41]. In addition, several temporal regions, including the inferior temporal (IT), entorhinal (ENT), and transverse temporal (TT), regions of ADHD patients manifested obviously reduced nodal efficiency. Increasing evidence has demonstrated the temporal lobe has a key role in ADHD. Furthermore, temporal lobe dysfunction might be associated with behavioral problems with response variability of ADHD patients [42], and the right temporal lobe is a highly affected locus in ADHD [43]. The precise neurobiological mechanism in the temporal regions and its influence on ADHD remains unclear; however, our results suggest that research on ADHD should expand to less studied brain areas, such as the temporal lobe.

In contrast, three brain regions were observed to have a notable increase in nodal efficiency in the ADHD group. These findings were in accordance with previous studies showing ADHD-related abnormalities in these regions. Tomasi [44] reported the functional connectivity density (FCD) of the superior frontal cortex was increased in ADHD children and had a positive correlation relationship with hyperactivity and impulsive behavior. Fisher [45] reported the EEG activity of ADHD subjects was increased in the paracentral lobule cortex. In summary, these results suggest that regions with abnormal nodal efficiency in brain structural networks are significantly affected and altered by ADHD.

The correlations between cortical thicknesses of brain regions and clinical symptoms of ADHD (ADHD_Index) were also assessed in this study. We found that the cortical thicknesses of many regions were negatively correlated with the ADHD_Index (Table 5). Because the ADHD_Index reflects the level of ADHD, a higher ADHD_Index score indicates more serious ADHD. The results of our study indicated that the greater ADHD symptom severity, the thinner the cortical thickness in the related brain regions. Interestingly, our results also identified that regions (left inferior temporal, left orbitofrontal, right medial orbitofrontal, and bilateral superior frontal) with a thinner cortical thickness in ADHD patients (Table 2) were associated with the severity of ADHD symptoms (Table 5). Furthermore, abnormal nodal efficiency (Figure 5 and Table 4) indicated that these five brain regions with thinner cortical thickness have a major role in the pathogenesis of ADHD. It was previously suggested that the above-mentioned regions were critical for response inhibition and executive function; thus, in future studies, quantitative morphological changes in these five regions might be promising measures for the diagnosis of ADHD in the clinic.

In conclusion, the current study observed abnormal cortical thickness and correlations in ADHD. Furthermore, our work revealed an extensive range of distribution changes in the brain anatomical networks and in the topological structure of the brain networks (which shifted to regular networks) in ADHD children. We also identified abnormal brain regions in the structural networks and confirmed altered morphologic features in these regions were associated with ADHD. These findings, consistent with previous ADHD studies using functional and structural imaging data, might aid our understanding of neural network disorders in ADHD, and provide evidence for the relationship between ADHD and cerebral disorder. However, because the correlation coefficient of cortical thickness is a statistical value, the ADHD and normal control groups could only construct one brain structural network, respectively. Therefore, the present study did not analyze the correlation between network properties (global and local efficiency as well as nodal efficiency) and ADHD_index or cortical thickness. Further studies should be carried out to analyze this issue. In addition, more accurate brain template should be used, and female pediatric patients should be recruited in future studies.

## MATERIALS AND METHODS

### Subjects

Eighty children (40 ADHD and 40 typically-developing controls) participated in the experiment (ADHD-200: Peking University; http://fcon_1000.projects.nitrc.org/indi/adhd200/). The ADHD patients were recruited from the Institute of Mental Health, Peking University. Normal controls were recruited from a local school. The age, gender balance, and IQ of the two groups are shown in Table 1. All subjects were right handed; had no lifetime history of neurological disease, head trauma, or other psychical illness; had full-scale IQ scores above 80 (Wechsler Intelligence Scale for Chinese Children-Revised, WISCC-R). The Computerized Diagnostic Interview Schedule IV (C-DIS-IV), which is based on DSM-IV, was used to diagnose ADHD. The ADHD Rating Scale (ADHD-RS) IV was employed to provide dimensional measures of ADHD symptoms. After diagnostic screening, all of the ADHD children recruited in this study were type of ADHD-Inattentive. The MINI Kid (Mini International Neuropsychiatric Interview) assessment was employed to ascertain the control group was healthy. The ethics committees of Peking University approved the study. According to the Declaration of Helsinki, written informed consent was obtained from the parents of all participants. Parents and teachers were the informant in diagnosis.

### MRI acquisition

MRI scans were performed on a Siemens Trio 3-T scanner (Siemens, Erlangen, Germany). All participants lay supine with a cushion and thermoplastic mask to reduce head movement effects. T1 images were collected for each subject with T1-weighted magnetization prepared rapid gradient-echo (MP-RAGE) sequences: sagittal 3D fast field echo scan with 128 slices, matrix = 256 × 256, time repetition [TR] = 2,530 ms, time inversion [TI] = 1,100 ms, thickness/gap = 1.33/0 mm, time echo [TE] = 3.39 ms, flip angle = 7°, and field of view = 256 mm × 256 mm.

### Measurements of cortical thickness

A 3D reconstruction of brain structure and brain region segmentation was performed using FreeSurfer software (http://surfer.nmr.mgh.harvard.edu/) on the Linux system. Using a 9-parameter linear transformation [46], the original MRI scans were registered into stereotaxic space [47]. Then, utilizing Non-parametric Non-uniform intensity Normalization algorithms [48], the images were corrected without artifacts. The corrected and registered images were used to reconstruct a highly accurate brain model and further divided into cerebrospinal fluid, white matter, and gray matter [49]. The pial, gray and white matter, and surfaces were then extracted from each volume of MRI [50] and the gray matter thickness was computed at any point in the cortical cortex. The surface reconstruction of each subject was later transformed into to a sphere with minimal distortion. The surfaces (sulcal/gyral) were then aligned with an average, canonical surface [51]. Figure 1A shows the different cortical thickness maps of the two groups. The present study used the Desikan-Killiany template (aparc.annot) in FreeSurfer [52] to parcellate the brain region. The Desikan-Killiany template includes 68 separate anatomical cortical regions of interest (34 in each hemisphere, Figure 1B and Appendix A), and has been widely used in segmentation of the cerebral cortex of adolescents and children [53, 54]. Finally, the cortical thickness for each region of interest was determined as the average thickness of all region vertices.

### Anatomical connectivity matrix and brain network construction

To measure the anatomical connectivity between brain regions, linear regression was applied at each region to remove several sources of influence (mean overall cortical thickness, age, and IQ) of cortical thickness; the residuals of this regression were then used instead of the original mean cortical thickness of the corresponding regions. Pearson's correlation coefficients between the residuals of each pair of the 68 regions were calculated to produce a symmetric correlation matrix for each subject (68 × 68), where the values of the diagonal were neglected (Figure 1C). Finally, the correlation matrix for each group was processed into a binarized matrix through the threshold.

This study utilized an undirected binary graph to explore the features of brain structural networks. The symmetric correlation matrices were converted to a binary graph by a threshold (Figure 1D). In the present study, network cost was employed to measure the threshold because it provided a physiologically significant description of a network's performance [36]. Network cost, *C*_*G*_, which is important for network efficiency, measures the cost to build a network. It is defined as follows:

$$C_{G}=\frac{K}{N(N-1)/2}$$ (1)

Note that *K* and *N* are the sum of edges (regions undirected connections) and nodes (each brain region) in graph *G*. *N (N-1)/2* is the number of all the possible edges in the graph. Given that there is no definite way to choose the precise threshold, we investigated a wide range of values of threshold. Here, the range of cost threshold was set an empirical range of values (from 0.05 to 0.4, step 0.005) to make the resulting matrices have sparse properties, and the small-world attributes estimable [8, 9, 17, 36]. Then we explored the differences in network features between the two groups at each cost value.

### Calculation of the network characteristic parameters

Optimized networks are defined as a high clustering coefficient and a low shortest path length; such networks are called small-world networks [7]. However, in recent small-world investigations, efficiency measurement has been a more effective method to analyze the network of local and global behavior and process the disconnected or non-sparse graphs [17, 33, 36]. In the present study, we explored brain structural networks in ADHD and normal control children using efficiency measures.

The efficiency of graph G is computed as follows:

$$E(G)=\frac{1}{N(N-1)}\sum_{i\neq j\in G}\frac{1}{L_{i,j}}$$ (2)

Note that *L*_*i, j*_ is the shortest path length between any two nodes *i* and *j*. When *G* indicates an entire network, *E (G)* measures the global efficiency. *E*_*glob*_ *(G)* is a global characteristic that evaluates the efficiency of transmission of information in the network. When considering a subgraph of *G*, *E* (*G*_*i*_) is the local efficiency of the local network *G*_*i*_ (*G*_*i*_ consists of the nearest neighbors of node *i*) and measures the local network information transmission efficiency. Therefore, the local efficiency of the whole network is defined as:

$$E_{loc}(G)=\frac{1}{N}\sum_{i\in G}G_{i}$$ (3)

where *E*_*loc*_ *(G)* is the mean efficiency *E (G_*i*_*) of all subgraphs involved in the graph [33].

In addition, we also performed regional nodal efficiency measurements, defined as:

$$E_{nodal}(G,i)=\frac{1}{N-1}\sum_{j\in G}\frac{1}{L_{i,j}}$$ (4)

where *E*_*nodal*_ *(G, i)* represents the exchange efficiency between a node *i* and all the other nodes in graph *G*.

Generally, statistical comparisons of small-world properties require comparable random and regular networks [7]. The theoretical networks are different from the experimental networks in the present study and thus cannot supply an effective contrast for our research of networks. Therefore, we also generated regular and random networks that maintained the same number of edges and nodes precisely. Then, we compared the efficiency of *G* with that of a regular network (*G*_*reg*_) and a random network (*G*_*rand*_). If *E*_*loc*_ (*G*_*rand*_) < *E*_*loc*_ *(G)* < *E*_*loc*_ (*G*_*reg*_) and *E*_*glob*_ (*G*_*reg*_) < *E*_*glob*_ *(G)* < *E*_*glob*_ (*G*_*rand*_), the research graph *G* is designated as an optimal small-world network [36].

### Statistical analysis

The Statistical Package for Social Studies (SPSS, version 13.0; SPSS, Inc., USA) and MATLAB (The MathWorks, Natick, MA, USA) were used for all statistical analyses.

To test the differences in cortical thickness, a two - groups (ADHD vs. Control) * 1 condition (thickness) analysis of variance (ANOVA) was performed for different cortical Regions of Interest (ROIs). Before this analysis, a linear regression was used to remove other effects (mean overall cortical thickness, age, and IQ) of cortical thickness; the residuals were used as the substitute for the original ROIs mean cortical thickness. *P* values < 0.05 were considered to indicate statistical significance.

To investigate whether the interregional correlation of cortical thickness between the two groups was significantly different, the present study used Fisher's *r*-to-*z* transform to convert the correlation coefficients to *z* values. Then, we compared these transformed z values to investigate the significance of the between-group differences in correlations. The present study used the non-parameter permutation test [55] of 5000 times to compare the correlation measures. Considering the correction for multiple comparisons, the FDR (false discovery rate) method was applied. A value of *P* < 0.05 was considered statistically significant.

Because of the calculation characteristics of the brain structural network, the present study used the non-parameter permutation test [55] of 5000 times to compare the global efficiency (*E*glob *(G)*), local efficiency (*E*loc *(G)*), and regional nodal efficiency (*E*nodal *(G, i)*) at each cost value to assess the differences of small-world topological features between the two groups. Each distribution adopted the 95 percentile point as the threshold, and the probability of a type I error was 0.05 (one-tailed test).

Spearman's test was performed to analyze correlations between the average cortex thickness and clinical symptoms of ADHD (ADHD_Index in ADHD 200: Peking University). The significance value was set as *P* < 0.05 for this correlation analysis.

## SUPPLEMENTARY MATERIALS TABLES


