# Supplementary material for: Altered brain structural networks in attention deficit/hyperactivity disorder children revealed by cortical thickness
Source: Oncotarget. 2017 Jan 18;8(27):44785–99. doi: 10.18632/oncotarget.14734 (PMC5546518; doi:10.18632/oncotarget.14734)
Supplement: Supplementary file 1 [file oncotarget-08-44785-s001.pdf]

Altered brain structural networks in attention deficit/ hyperactivity disorder children revealed by cortical thickness

Table A. Anatomical cortical regions of interest in an human brain

Region - Abbrevation	Region - Abbrevation
Bank of the superior temporal sulcus - BSTS	Pars orbitalis (Inferior frontal) - PORB
Caudal anterior cingulate - CAC	Pars triangularis (Inferior frontal) – PTRI
Caudal middle frontal - CMF	Pericalcarine – PERI
Cuneus – CUN	Postcentral gyrus - PSTC
Entorhinal – ENT	Posterior cingulate - PC
Fusiform gyrus – FUSI	Precentral gyrus - PREC
Inferior parietal – IP	Precuneus – PCUN
Inferior temporal – IT	Rostral anterior cingulate - RAC
Isthmus of the cingulate – ISTC	Insula – INS
Lateral occipital – LOCC	Rostral middle frontal - RMF
Lateral orbitofrontal - LOF	Superior frontal – SF
Lingual gyrus – LING	Superior parietal - SP
Medial orbitofrontal - MOF	Superior temporal - ST
Middle temporal – MT	Supramarginal – SMAR
Parahippocampal – PHG	Frontal pole – FP
Paracentral lobule - PARC	Temporal pole – TP
Pars opercularis (Inferior frontal) – POPE	Transverse temporal – TT
